# Current status of delirium assessment tools in the intensive care unit: a prospective multicenter observational survey

**DOI:** 10.1038/s41598-022-06106-w

**Published:** 2022-02-09

**Authors:** Kenzo Ishii, Kosuke Kuroda, Chika Tokura, Masaaki Michida, Kentaro Sugimoto, Tetsufumi Sato, Tomoki Ishikawa, Shingo Hagioka, Nobuki Manabe, Toshiaki Kurasako, Takashi Goto, Masakazu Kimura, Kazuharu Sunami, Kazuyoshi Inoue, Takashi Tsukiji, Takeshi Yasukawa, Satoshi Nogami, Mitsunori Tsukioki, Daisuke Okabe, Masaaki Tanino, Hiroshi Morimatsu

**Affiliations:** 1 Department of Anesthesiology, Intensive Care Unit, Fukuyama City Hospital, 5-23-1 Zao-cho, Fukuyama, Hiroshima 721-8511 Japan; 2 Department of Anesthesiology and Resuscitology, Okayama University Graduate School of Medicine, Dentistry and Pharmaceutical Sciences, Okayama, Japan; 3 Department of Anesthesia, Kagawa Rosai Hospital, Marugame, Kagawa Japan; 4 Department of Anesthesiology and Intensive Care Medicine, Kawasaki Medical School General Medical Center, Okayama, Japan; 5 Department of Anesthesia, Chikamori Hospital, Kochi, Japan; 6 Department of Anesthesia and Intensive Care, National Cancer Center Hospital, Tokyo, Japan; 7 Department of Anesthesia, Okayama Red Cross General Hospital, Okayama, Japan; 8 Department of Anesthesia, Tsuyama Chuo Hospital, Tsuyama, Okayama Japan; 9 Department of Anesthesia, Saiseikai Imabari Hospital, Imabari, Ehime Japan; 10 Department of Anesthesiology, Japanese Red Cross Society Himeji Hospital, Himeji, Hyogo Japan; 11 Department of Anesthesia, Hiroshima City Hiroshima Citizens Hospital, Hiroshima, Japan; 12 Department of Anesthesia, Okayama City Hospital, Okayama, Japan; 13 Department of Internal Medicine, Okayama Kyoritsu Hospital, Okayama, Japan; 14 Department of Anesthesia, Kagawa Prefectural Central Hospital, Takamatsu, Kagawa Japan; 15 Department of Anesthesia, Takasago Municipal Hospital, Takasago, Hyogo Japan; 16 Department of Anesthesia, Okayama Kyokuto Hospital, Okayama, Japan; 17 Department of Anesthesia, National Hospital Organization Okayama Medical Center, Okayama, Japan; 18 Department of Anesthesia, Onomichi Municipal Hospital, Onomichi, Hiroshima Japan; 19 Department of Anesthesia, Himeji St. Mary’s Hospital, Himeji, Hyogo Japan; 20 Department of Anesthesiology and Intensive Care Medicine, Kawasaki Medical School Hospital, Kurashiki, Okayama Japan

**Keywords:** Diseases, Medical research, Neurology, Risk factors

## Abstract

Delirium is a critical challenge in the intensive care unit (ICU) or high care unit (HCU) setting and is associated with poor outcomes. There is not much literature on how many patients in this setting are assessed for delirium and what tools are used. This study investigated the status of delirium assessment tools of patients in the ICU/HCU. We conducted a multicenter prospective observational study among 20 institutions. Data for patients who were admitted to and discharged from the ICU/HCU during a 1-month study period were collected from each institution using a survey sheet. The primary outcome was the usage rate of delirium assessment tools on an institution- and patient-basis. Secondary outcomes were the delirium prevalence assessed by each institution’s assessment tool, comparison of delirium prevalence between delirium assessment tools, delirium prevalence at the end of ICH/HCU stay, and the relationship between potential factors related to delirium and the development of delirium. Result showed that 95% of institutions used the Intensive Care Delirium Screening Checklist (ICDSC) or the Confusion Assessment Method for the ICU (CAM-ICU) to assess delirium in their ICU/HCU, and the remaining one used another assessment scale. The usage rate (at least once during the ICU/HCU stay) of the ICDSC and the CAM-ICU among individual patients were 64.5% and 25.1%, and only 8.2% of enrolled patients were not assessed by any delirium assessment tool. The prevalence of delirium during ICU/HCU stay was 17.9%, and the prevalence of delirium at the end of the ICU/HCU stay was 5.9%. In conclusion, all institutions used delirium assessment tools in the ICU/HCU, and most patients received delirium assessment. The prevalence of delirium was 17.9%, and two-thirds of patients had recovered at discharge from ICU/HCU.

**Trial registration number**: UMIN000037834.

## Introduction

Delirium is a critical challenge in the intensive care unit (ICU). Delirium is associated with poor outcomes, including high cost^1,2^, high mortality^2,3^, longer duration of mechanical ventilation (MV)^1,3,4^, longer duration of ICU stay^1,3^, and longer duration of hospitalization^2,4^. Many patients (19.6–81.7%) experience delirium during their stay in the ICU^3–10^. Previous studies highlighted the importance of assessing, preventing, and managing delirium in the ICU^1,2,11,12^. However, prevention and treatment of delirium may be complicated by baseline risk factors among ICU patients, including preexisting dementia, history of hypertension or alcoholism, and high severity of illness at admission^2^. According to the “Clinical Practice Guidelines for the Prevention and Management of Pain, Agitation/Sedation, Delirium, Immobility, and Sleep Disruption in Adult Patients in the ICU” (2018 PADIS guidelines), these risk factors can be divided into two categories: modifiable and nonmodifiable^1^. As it is difficult to control nonmodifiable risk factors among ICU patients, prevention of delirium is limited.

The earlier “Clinical Practice Guidelines for the Management of Pain, Agitation, and Delirium in Adult Patients in the Intensive Care Unit” (2013 PAD guidelines)^2^ for the assessment of delirium in ICU patients recommended using valid tools to assess delirium, such as the Intensive Care Delirium Screening Checklist (ICDSC) or the Confusion Assessment Method for the ICU (CAM-ICU). A good practice statement in the 2018 PADIS guidelines indicates that critically ill adults should be regularly assessed for delirium using a valid tool^1^. Despite delirium evaluation being emphasized in these guidelines, implementation rates of delirium evaluation tools are relatively low in some countries (3–34.04%)^13–15^. For example, reported implementation rates were based on nationwide surveys of ICU physicians from India (480 physicians)^13^, China (1011 physicians)^14^, and Poland (165 physicians)^15^. Also, a multinational survey reported that 65% of 101 participating ICUs reported monitoring for delirium in clinical routine, and 44% reported the use of a validated delirium score on the institutional level. In contrast, they reported that 73% of 868 included patients were not monitored with a validated score on patient level^16^. This also means that the high usage rate of delirium assessment tools at the institutional level does not necessarily mean that many patients are being assessed for delirium. Furthermore, in Japan, it is unclear how many institutions use these tools and how many patients are assessed for delirium.

Therefore, we focused on assessment of delirium in critically ill patients in Japan. Specifically, this study aimed to investigate the actual status of delirium evaluation for patients admitted to the ICU or high care unit (HCU). We focused on institution-based surveys and patient-based surveys in the participating institutions and collected data from 20 institutions for over 1000 patients.

## Methods

We conducted a multicenter prospective observational study among institutions in the Okayama Research Investigation Organizing Network (ORION) group, consisting of about 50 research institutes around Okayama, Japan. This study was approved by the Central Institutional Review Board at Okayama University Graduate School of Medicine, Dentistry and Pharmaceutical Sciences and the Okayama University Hospital Ethics Committee (approval no. Ken-1905-033; June 7, 2019). Two of the participating institutions conducted independent ethical reviews. The need to obtain informed consent from patients was waived because this study used an observational design. However, we provided an opportunity for patients to opt-out of this study. This study was performed in accordance with the Declaration of Helsinki. The authors declare no conflicts of interest.

We collected data from the ICU/HCU in the participating institutions for a 1-month period (September or November 2019). We included all patients who were admitted to and discharged from the ICU/HCU during the study period. We did not exclude children in this study.

Data were collected using two types of survey sheets, one for each institution and one for each patient. The representative of each institution answered the data on the institution sheet. The data on the survey sheet for each patient was collected from the patient’s medical record and chart by the institution’s staff. Data collected from each institution included the total number of hospital beds, number of ICU/HCU beds, and numbers of ICU physicians and nurses. Each institution also reported which delirium assessment tool had been used (i.e., ICDSC, CAM-ICU, Diagnostic and Statistical Manual of Mental Disorders, Fifth Edition [DSM-5], psychiatrist-based assessment, or others). If no tool had been introduced, they reported “None”.

The patient-based survey collected data including: patients’ baseline characteristics (age, gender, duration of ICU/HCU stay, Acute Physiology and Chronic Health Evaluation [APACHE] II score, and Sequential Organ Failure Assessment [SOFA] score), admission route to the ICU/HCU (after scheduled operation, after emergency operation, in-hospital emergency, or out of hospital emergency), treatment (MV, high flow nasal cannula [HFNC], renal replacement therapy [RRT], and mechanical support for circulation), preexisting factors related to delirium at admission (dementia, use of sleep or psychological drugs, history of delirium, hypertension, and alcoholism). All of these data were collected from the patient's medical records. To make this study feasible, we did not diagnose dementia in this study nor specify sleep or psychological drugs in detail.

We also collected data related to delirium assessment tools, including the delirium assessment tool used for each patient at least once during their ICU/HCU stay (ICDSC, CAM-ICU, DSM-5, psychiatrist, assessment based on clinical condition, and others), and the user and evaluator of the delirium assessment tools for each patient (ICU physician, physician in charge, psychiatrist, or nurse). We also recorded the development of delirium and its date and the presence of delirium at the end of their ICU / HCU stay. The definition of delirium used in this study was: (1) patient assessed by the ICDSC as scoring ≥ 4 at any time during their ICU/HCU stay; (2) patient assessed by the CAM-ICU as having delirium at any time during ICU/HCU stay; and (3) patient assessed with delirium by any method used at that institution at any time during ICU/HCU stay. If the institution used several tools (e.g., ICDSC or CAM-ICU) on the same patient, the patient was evaluated as delirium when at least one of those tools had a positive result.

This study’s primary outcome was the usage rate of delirium assessment tools on an institution- and patient-basis. The secondary outcomes were the delirium prevalence (using the above definition) as assessed by each institution’s assessment tool, comparison of delirium prevalence between delirium assessment tools, delirium prevalence at the end of the ICH/HCU stay, and the relationship between potential factors related to delirium and the development of delirium.

Each patient was surveyed using a data sheet with selected questions and a mark sheet. The questions (multi-choice) covered: treatment, preexisting factors related to delirium at admission, the delirium assessment tool used for each patient, and the user and evaluator of the delirium assessment tool for each patient.

We did not calculate the sample size needed for this study because we decided on an adequate study period in which each institution could collect data. However, before enrolling patients, we predicted that approximately 1300 patients would be enrolled in this study from the participating facilities. STATA (SE 15.1) was used to analyze the data. For the statistical analyses, we used chi-square tests for categorical data. Mann–Whitney U tests was used because each numerical variable was not distributed normally. The collected data were described using median and interquartile range (IQR) or percentages. We also used these two tests to calculate the odds ratio (OR).

## Results

In total, 20 institutions from the ORION group participated in this study. Of these 20 institutions, 16 facilities (80%) used the ICDSC, and five facilities (25%) used the CAM-ICU (Table 1). Either the ICDSC or the CAM-ICU (or both) were used in 95% of facilities, and the remaining one used another assessment scale (The Neelon and Champagne [NEECHAM] Confusion Scale). Therefore, no institution answered they did not assess delirium in their ICU/HCU.

**Table 1:** Number of institutions introducing each delirium assessment tool.

	ICDSC	CAM-ICU	DSM-5	Psychiatrist	Others	None*
Number (%)	16 (80)	5 (25)	2 (10)	5 (25)	2 (10)	0

*ICDSC* Intensive Care Delirium Screening Checklist, *CAM-ICU* Confusion Assessment Method for the Intensive Care Unit, *DSM-5* Diagnostic and Statistical Manual of Mental Disorders, Fifth Edition.

Data are n (%).

The percentages add up to over 100% because multiple selections were possible.

*Institutions that had never assessed delirium.

During the study period, 1382 patients were newly admitted to and discharged from the ICU/HCU in the participating institutions. Of these, we excluded 172 patients because of missing data (no survey sheet was submitted) and collected and analyzed data for 1210 patients (Table 2). The data inclusion rate of this study was 87.6%.

**Table 2:** Basic characteristics of each institution, number of registered patients, and submission rate.

Institutions	Total number of beds	Number of ICU/HCU beds	Number of ICU physicians	Number of nurses	Total number of ICU/HCU inpatients during the study period	Number of registered patients	Data submission rate (%)
A	191	11	0	21	73	67	91.8
B	213	12	0	49	36	36	100
C	214	12	3	27	33	32	97
D	290	8	0	23	45	30	66.7
E	318	8	0	17	41	40	97.6
F	360	4	0	16	30	28	93.3
G	400	14	0	35	50	46	92.0
H	404	16	0	86	101	101	100
I	500	12	1	44	77	77	100
J	506	12	1	43	83	83	100
K	512	18	0	43	97	83	85.6
L	515	20	2	55	121	69	57
M	533	8	1	31	44	39	88.6
N	560	10	2	40	72	66	91.7
O	570	8	0	33	87	79	90.8
P	609	6	1	27	36	30	83.3
Q	647	22	1	79	101	86	85.1
R	743	14	1	46	77	65	84.4
S	855	22	2	76	150	133	88.7
T	1154	11	0	40	28	20	71.4
Total		248			**1382**	**1210**	87.6

*HCU* high care unit, *ICU* intensive care unit, A, B, C…, S, T represent participating institutional (anonymous).

We included 1382 patients who were newly admitted to and discharged from the ICU/HCU, but excluded 172 patients because of missing data; therefore, we collected and analyzed data for 1210 patients.

Significant values are in bold.

Patients’ baseline characteristics are shown in Table 3. The median (IQR) age was 71 (61, 81) years (minimum 0, maximum 103 years old), and 56% of patients were men. The preexisting factors related to delirium at admission were dementia (9.0%), use of sleep or psychological drugs (17.4%), history of delirium (4.4%), history of hypertension (46.0%), and history of alcoholism (1.4%). All data from individual patients were collected, except for APACHE II score and SOFA score because it was not mandatory for the institutions to calculate those two scores specifically for this study. Data for the APACHE II and SOFA scores were available for 87.1% and 87.0% of patients, respectively. The median (IQR) APACHE II score was 12 (8, 17), and that for the SOFA score was 3 (1, 5).

**Table 3:** Baseline characteristics of all patients, and comparison between delirium group and non-delirium group.

	Total patients	Delirium group	Non-delirium group	*P* value	
Number of patients	n = 1210	n = 217	n = 894		(n = 1111)
Age, years	72 (61, 81)	80 (71, 87)	70 (58, 78)	< 0.001	*
Gender (% men)	56	57.6	56.2	0.7	
Duration of ICU/HCU stay, days	2 (2, 4)	4 (3, 7)	2 (2, 4)	< 0.001	*
APACHE II score	12 (8, 17)	16.5 (12, 21)	12 (8, 16)	< 0.001	* (n = 969)
SOFA score	3 (1, 5)	4 (2, 7)	2 (1, 4)	< 0.001	* (n = 960)
**Admission route**					
After scheduled operation (%)	48.4	24.9	56.9	< 0.001	*
After emergency operation (%)	10.6	11.1	11.3		
In-hospital emergency (%)	8.4	14.3	6.3		
Out of hospital emergency (%)	32.6	49.8	25.5		
**Treatment**					
Mechanical ventilation (%)	24.3	35	22	< 0.001	*
High flow nasal cannula (%)	4.9	6.9	4.6	0.16	
Renal replacement therapy (%)	4.2	7.8	3.5	0.005	*
Mechanical support for circulation (%)	2.1	3.7	1.7	0.07	
**Preexisting factors related to delirium at admission**					
Dementia (%)	9.0	26.7	4.6	< 0.001	*
Use of sleep or psychological drugs (%)	17.4	25.8	15.8	< 0.001	*
History of delirium (%)	4.4	12.9	2.5	< 0.001	*
History of hypertension (%)	46	49.8	45.3	0.24	
History of alcoholism (%)	1.4	2.3	1.3	0.3	
Patients without these preexisting factors (%)	39.3	23	42.8	< 0.001	*
**Delirium assessment tool used for each patient**					
ICDSC (%)	64.5	77	67.9	< 0.001	*
CAM-ICU (%)	25	26.7	27.4	0.84	
DSM-5 (%)	0	0	0		
Psychiatrist (%)	1.2	5.5	0.2	< 0.001	*
Assessment based on clinical condition (%)	10	15.2	9.8	0.02	*
Others (%)	8.1	5.1	9.5	0.04	*
**User of delirium assessment tools**					
ICU physician (%)	7	12.4	6	0.001	*
Physician in charge (%)	4.4	6	4.5	0.35	
Psychiatrist (%)	1.2	5.5	0.3	< 0.001	*
Nurse (%)	91.0	98.2	97.7	0.65	
**Evaluator of delirium assessment tools**					
ICU physician (%)	26.0	28.6	27.6	0.78	
Physician in charge (%)	5.3	7.4	5.4	0.26	
Psychiatrist (%)	1.5	6.5	0.4	< 0.001	*
Nurse (%)	83.9	93.1	89.7	0.13	

*p < 0.05, *APACHE* Acute Physiology and Chronic Health Evaluation, *SOFA* Sequential Organ Failure Assessment, *ICDSC* Intensive Care Delirium Screening Checklist, *CAM-ICU* Confusion Assessment Method for the Intensive Care Unit, *DSM-5* Diagnostic and Statistical Manual of Mental Disorders, Fifth Edition, *ICU* intensive care unit.

Data are median (interquartile range) or percentage.

Analysis of tool used for individual patients (at least once during the ICU/HCU stay) showed the ICDSC was used for 64.5% of patients, and the CAM-ICU for 25.1% of patients. In total, 8.2% of patients were not assessed by any delirium assessment tool. We found that 17.9% of the enrolled patients had delirium during their ICU/HCU stay. The median length of ICU/HCU stay until the onset of delirium was 1 day. Patients were divided into two groups (delirium group and non-delirium group), excluding the 8.2% (n = 99) of patients who were not evaluated. We compared the basic characteristics and survey data between the two groups (Table 3). The median age (IQR) was 80 (71, 87) years in the delirium group and 70 (58, 78) years in the non-delirium group (*p* < 0.001). The median (IQR) duration of ICU/HCU stay was significantly longer in the delirium group than the non-delirium group: 4 (3, 7) days vs. 2 (2, 4) days (*p* < 0.001). In addition, compared with the non-delirium group, the delirium group had significantly higher median (IQR) APACHE II scores (16.5 [12, 21] vs. 12 [8, 16]; *p* < 0.001) and SOFA scores (4 [2, 7] vs. 2 [1, 4]; *p* < 0.001). We also observed significant differences between the two groups in admission route (*p* < 0.001), MV (35.0% vs. 22.0%, *p* < 0.001), RRT (7.8% vs. 3.5%, *p* = 0.005), dementia (26.7% vs. 4.6%, *p* < 0.001), use of sleep or psychological drugs (25.8% vs. 15.8% *p* < 0.001), and history of delirium (12.9% vs. 2.5%, *p* < 0.001).

We found that 5.9% of the enrolled patients had delirium at the time of discharge from the ICU/HCU. We therefore divided patients assessed as having delirium during their ICU/HCU stay into two groups (those with delirium at the end of the ICU/HCU stay and those without delirium at the end of the ICU/HCU stay). We excluded 12 patients who were not evaluated at the end of their ICU/HCU stay. Comparison of basic characteristics and survey data between the two groups (Table 4) showed significant differences in age (83 [78, 89.5] years vs. 77 [70, 85.75] years, *p* < 0.001) and dementia (45.1% vs. 18.7%, *p* < 0.001).

**Table 4:** Comparison between patients with and without delirium at the end of ICU stay in patients assessed with delirium during ICU stay.

	With delirium at the end of ICU/HCU stay	Without delirium at the end of ICU/HCU stay	*P* value	
Number of patients	n = 71	n = 134		(n = 205)
Age, years	83 (78, 89.5)	77 (70, 85.75)	< 0.001	*
Gender (% men)	50.1	62.7	0.1	
Duration of ICU/HCU stay, days	4 (2, 7)	4 (3, 6.75)	0.75	
Duration from admission to delirium	1 (0, 1.5)	1 (0, 2)	0.77	
APACHE II score	15 (11, 21)	16 (12, 21)	0.86	(n = 182)
SOFA score	5 (3, 6)	4 (2, 7.75)	0.90	(n = 180)
**Admission route**				
After scheduled operation (%)	22.5	27.6	0.65	
After emergency operation (%)	9.9	11.2		
In-hospital emergency (%)	11.3	11.2		
Out of hospital emergency (%)	56.3	47		
**Treatment**				
Mechanical ventilation (%)	35.2	35.1	0.98	
High flow nasal cannula (%)	4.2	8.2	0.28	
Renal replacement therapy (%)	5.6	9	0.4	
Mechanical support for circulation (%)	2.8	3.7	0.73	
**Preexisting factors related to delirium at admission**				
Dementia (%)	45.1	18.7	< 0.001	*
Use of sleep or psychological drugs (%)	23.9	26.9	0.65	
History of delirium (%)	18.3	9.7	0.08	
History of hypertension (%)	46.5	51.5	0.49	
History of alcoholism (%)	0	3.7	0.1	
Patients without these preexisting factors (%)	15.5	26.9	0.07	
**Delirium assessment tool used for each patient**				
ICDSC (%)	76.1	80.6	0.45	
CAM-ICU (%)	25.4	24.6	0.91	
DSM-5 (%)	0	0		
Psychiatrist (%)	11.3	1.5	0.002	*
Assessment based on clinical condition (%)	22.5	11.9	0.047	*
Others (%)	5.6	3.7	0.53	

*p < 0.05, *ICU* intensive care unit, *APACHE* Acute Physiology and Chronic Health Evaluation, *SOFA* Sequential Organ Failure Assessment, *ICDSC* Intensive Care Delirium Screening Checklist, *CAM-ICU* Confusion Assessment Method for the Intensive Care Unit, *DSM-5* Diagnostic and Statistical Manual of Mental Disorders, Fifth Edition.

Data are median (interquartile range) or percentage.

**Figure 1:**
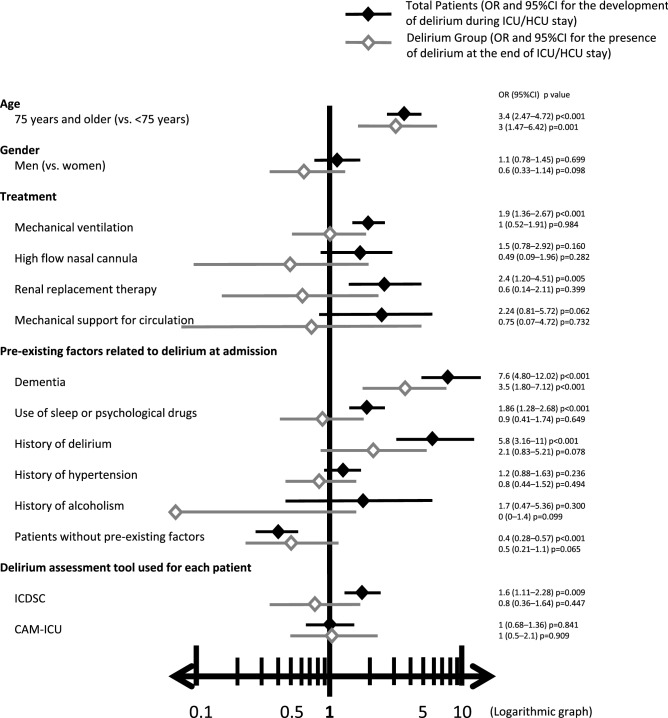
Odds ratio (OR) and 95% confidence interval (95%CI) for each potential factor related to delirium in total patients (development of delirium during ICU/HCU stay) and the delirium group (presence of delirium at the end of ICU/HCU stay). *ICU* intensive care unit, *ICDSC* Intensive Care Delirium Screening Checklist, *CAM-ICU* Confusion Assessment Method for the Intensive Care Unit.

Furthermore, we calculated the odds ratio (OR) and 95% confidence interval (95%CI) for each potential factor related to delirium for two categories: total patients (OR and 95%CI of the development of delirium during ICU/HCU stay) and the delirium group (OR and 95%CI of the presence of delirium at the end of ICU/HCU stay) (Fig. 1). This allowed comparison of the OR for delirium between these two categories. Patients with older age and those with dementia were significantly relevant for delirium in both categories. However, although use of sleep or psychological drugs, and history of delirium had a significant relationship in the analysis with total patients, there were no significant differences in the analysis using the delirium group. In terms of treatment, MV (significant), RRT (significant), HFNC (trend), and mechanical support for circulation (trend) showed higher OR in the total patients analysis. However, these four factors were not significantly relevant at the end of ICU/HCU stay.

## Discussion

This study aimed to clarify current use of delirium assessment tools in ICU/HCU settings in Japan, along with the prevalence of delirium and its relationship with potential factors related to delirium during ICU/HCU stay and at discharge. The results showed that most participating facilities used the ICDSC or the CAM-ICU as a delirium assessment tool. The ICDSC was used for 64.5% of patients, and the CAM-ICU for 25.1% of patients. In total, 8.2% of patients were not assessed by any delirium assessment tool. In this study, the prevalence of delirium during ICU/HCU stay was 17.9%, and the prevalence of delirium at the end of the ICU/HCU stay was 5.9%. Many factors had significant relationships with delirium during ICU/HCU stay (Table 3). However, only older age and dementia were significantly relevant factors for delirium at the end of ICU/HCU stay (Table 4).

We aimed to clarify the current use of delirium assessment tools in the ICU/HCU setting. The results showed that 95% of all participating facilities used the ICDSC or the CAM-ICU as a delirium assessment tool, and the remaining one used another assessment scale (NEECHAM Confusion Scale). Compared with other studies from India (usage rate of CAM-ICU: 20%)^13^, China (delirium scale: 34.04%)^14^, and Poland (ICDSC: 0%, CAM-ICU: 3.03%)^15^, our data showed a higher usage rate of delirium assessment tools than expected. The two major tools used were the ICDSC and the CAM-ICU, with the usage rate of ICDSC being higher than that of the CAM-ICU in institutions (80% vs. 25%) and for individual patients (64.5% vs. 25.1%) (at least once during the ICU/HCU stay). In total, 8.2% of patients were not assessed by any delirium assessment tool. A multinational survey reported in 2014 that 65% of participated ICUs monitored for delirium, and 44% used validated delirium scores on the institutional level. In contrast, they reported that 73% of included patients were not monitored with a validated score on patient level^16^. Comparing these data, we found in this study that more institutions had already introduced delirium assessment tools, and more patients were being assessed for delirium by any assessment tools. These improvements in the use of delirium assessment tools might be due to the announcement of the 2018 PADIS guideline^1^ or earlier 2013 PAD guidelines^2^. Some previous studies compared the accuracy^17,18^ and agreement^19–21^ between these tools. However, determining which tool was better to use in the ICU/HCU setting was outside the scope of this study. In addition, our patient-based survey showed that few patients (8.2% of all patients) did not receive any delirium assessment. We considered this finding was novel in terms of understanding delirium assessment for patients in the Japanese ICU/HCU setting.

This study showed that the prevalence of delirium during ICU/HCU stay was 17.9%, and the prevalence of delirium at the end of the ICU/HCU stay was 5.9%. Previous studies reported the delirium incidence among critically ill adults was 19.6–81.7%^3–10^ and among critically ill children was 18.6–25%^22,23^. Our results on the prevalence of delirium were lower than in previous reports. We thought this was because we included all patients admitted to and discharged from the ICU/HCU during the study period. This study aimed to know the current status of delirium assessment tools, and our data included children to elders with many types of backgrounds. The median length until the development of delirium was 1 day, which showed that delirium might be developed in the early stages after admission to ICU/HCU. We also found that two-thirds of patients who experienced delirium had recovered at the end of their ICU/HCU stay.

We compared the potential factors related to delirium during ICU/HCU stay and at discharge (Fig. 1). Consistent with previous studies, many factors had significant relationships with delirium, including preexisting dementia^2^, history of hypertension^2^, history of alcoholism^2,5^, high severity of illness at admission^2,5,8^, age^5,9^, pain^9^, MV^4,22^, length of hospitalization^4^, use of benzodiazepines^5,22^, fever^8^, use of physical restraints^4,8,22^, and sleep deprivation^8^. We selected preexisting factors related to delirium as dementia, use of sleep or psychological drugs, history of hypertension, and history of alcoholism according to the 2013 PAD guidelines^2^. Among these preexisting factors we collected, we found that only older age and dementia were significantly relevant for delirium at the end of ICU/HCU stay. The 2018 PADIS guidelines^1^ divide risk factors for delirium into two categories: modifiable (e.g., benzodiazepine use and blood transfusions) and nonmodifiable (e.g., older age, dementia, prior coma, pre-ICU emergency surgery or trauma, and higher APACHE and the American Society of Anesthesiologists scores). Our data showed that relationships between delirium and modifiable factors such as MV, RRT, and use of sleep or psychological drugs had disappeared at the end of ICU/HCU stay. However, older age and dementia are nonmodifiable factors in the ICU/HCU setting, meaning these relevant factors to delirium remained throughout the ICU/HCU stay.

We found that most patients received delirium assessment at least once during their ICU/HCU stay. This finding may inform our subsequent study focused on treatment and/or prevention of delirium and may also improve delirium management in critically ill patients. A systematic review and meta-analysis^24^ reported the CAM-ICU was the delirium assessment tool most frequently used for research (65% of included studies). This data differed from our finding that use of the ICDSC was more common (ICDSC 64.5% vs. CAM-ICU 25.1% on a patient basis). Unfortunately, we did not collect why individual facilities had used these tools. We guessed that ICDSC might have been considered easy to introduce in clinical settings for ICU/HCU. However, other studies suggested that both the CAM-ICU and the ICDSC can be used as screening tools for diagnosing delirium in critically ill patients^2,25^. As it is important to assess delirium among critically ill patients, each institution can choose an appropriate delirium assessment tool.

This study had six limitations: the design (observational study), the definition of ICU, the sample size, the definition of delirium, data collection by co-authors, and data collection for preexisting medical conditions. First, this study was observational, meaning it was difficult to clarify causal relationships between possible risk factors and delirium. However, it was possible to discuss the relationship between potential risk factors and delirium. Second, we collected data from both ICUs and HCUs. We believe our inclusion of a variety of institutions increased the generalizability of our study and reflected the current situation of ICU/HCU in Japanese hospitals. Third, we did not calculate the required sample size before collecting data because we decided on a 1-month data collection period that would allow effective completion of this survey. However, we collected data for 1210 cases, and the data submission rate from all institutions was 87.6%. Therefore, we considered these data were sufficient and reliable to analyze. Fourth, we defined the development of delirium in this study as evaluation based on assessment tools that each institution already used. Therefore, our definition varied slightly between institutions, and this difference could have introduced bias in comparing and analyzing the prevalence and potential factors related to delirium. However, it was not possible to use the same definition across all institutions in this kind of survey. Fifth, data collection was carried out by representatives of the institution, including the co-authors of this study. This could be a bias in the data for this study. Finally, we collected preexisting conditions, including dementia and sleep or psychological drugs, from the patient's medical records, and we did not make a new diagnosis of dementia in this study. We did not specify sleep or psychological drugs in detail. This might be underestimated to detect these preexisting factors. However, we thought it was important to make this study feasible and decided not to collect the preexisting medical conditions in detail before starting this study.

In conclusion, all participating institutions had already introduced delirium assessment tools, and 95% of them used ICDSC and/or CAM-ICU. In addition, most patients who participated in this study received delirium assessment in ICUs/HCUs. The prevalence of delirium during the ICU/HCU stay was 17.9%, and two-thirds of these patients were recovered at the end of the ICU/HCU stay.
